# Reduced Health-Related Quality of Life in Elders with Frailty: A Cross-Sectional Study of Community-Dwelling Elders in Taiwan

**DOI:** 10.1371/journal.pone.0021841

**Published:** 2011-07-01

**Authors:** Cheng-Chieh Lin, Chia-Ing Li, Chiu-Kai Chang, Chiu-Shong Liu, Chih-Hsueh Lin, Nai-Hsin Meng, Yih-Dar Lee, Fei-Na Chen, Tsai-Chung Li

**Affiliations:** 1 Department of Family Medicine, China Medical University & Hospital, Taichung, Taiwan; 2 Medical Research, China Medical University & Hospital, Taichung, Taiwan; 3 Department of Family Medicine, College of Medicine, China Medical University & Hospital, Taichung, Taiwan; 4 Institute of Health Care Administration, College of Public Health, China Medical University & Hospital, Taichung, Taiwan; 5 Department of Healthcare Administration, College of Health Science, Asia University, Taichung, Taiwan; 6 Department of Physical Medicine and Rehabilitation, China Medical University & Hospital, Taichung, Taiwan; 7 Department of Psychiatry, Medical College, National Cheng-Kung University, Tainan, Taiwan; 8 Bristol-Myers Squibb (Taiwan) Ltd, Global Development & Medical Affair, Taipei, Taiwan; 9 School of Medicine, China Medical University & Hospital, Taichung, Taiwan; 10 Graduate Institute of Biostatistics & Chinese Medicine Science, China Medical University & Hospital, Taichung, Taiwan; 11 Biostatistics Center, China Medical University & Hospital, Taichung, Taiwan; Marienhospital Herne - University of Bochum, Germany

## Abstract

**Purpose:**

Exploring the domains and degrees of health-related quality of life (HRQOL) that are affected by the frailty of elders will help clinicians understand the impact of frailty. This association has not been investigated in community-dwelling elders. Therefore, we examined the domains and degree of HRQOL of elders with frailty in the community in Taiwan.

**Methods:**

A total of 933 subjects aged 65 years and over were recruited in 2009 from a metropolitan city in Taiwan. Using an adoption of the Fried criteria, frailty was defined by five components: shrinking, weakness, poor endurance and energy, slowness, and low physical activity level. HRQOL was assessed by the short form 36 (SF-36). The multiple linear regression model was used to test the independent effects of frailty on HRQOL.

**Results:**

After multivariate adjustment, elders without frailty reported significantly better health than did the pre-frail and frail elders on all scales, and the pre-frail elders reported better health than did the frail elders for all scales except the scales of role limitation due to physical and emotional problems and the Mental Component Summary (MCS). The significantly negative differences between frail and robust elders ranged from 3.58 points for the MCS to 22.92 points for the physical functioning scale. The magnitude of the effects of frail components was largest for poor endurance and energy, and next was for slowness. The percentages of the variations of these 10 scales explained by all factors in the models ranged from 11.1% (scale of role limitation due to emotional problems) to 49.1% (scale of bodily pain).

**Conclusions:**

Our study demonstrates that the disabilities in physical health inherent in frailty are linked to a reduction in HRQOL. Such an association between clinical measures and a generic measure of the HRQOL may offer clinicians new information to understand frailty and to conceptualize it within the broader context of disability.

## Introduction

Frailty is one of the greatest gerontological challenges faced by Taiwan because it has one of the fastest ageing populations in the world. Frailty has been defined as a multidimensional syndrome, and is characterized by the loss of reserves including energy, physical ability, cognition and health [1]–[3]. Frail elders are considered to be vulnerable to adverse health outcomes, including mortality, institutionalization, falls, and hospitalization [4]–[6]. The markers of frailty include age-associated declines in lean body mass, strength, endurance, balance, walking performance, and low activity [7]–[10].

The Short Form 36 (SF-36) assesses health concepts that represent basic human values relevant to everyone's functional status and well-being [11]–[12]. It assesses health-related quality of life (HRQOL) outcomes, which are composed of disability and discomfort components. Exploring the domains and degrees of functioning and well-being that are affected by the frailty of elders will help clinicians to understand the impact of frailty on functional status and well-being. Previous studies have investigated the impact of frailty on HRQOL, and findings have been reported for community-dwelling elders referred to an outpatient geriatric service [13], patients with heart failure [14], older adults with cardiometabolic risk factors [15], institutionalized older persons [16] or older Mexican Americans [17]. Although the effect of frailty had been examined in community-dwelling older adults in the Netherlands [18] or in Mexican Americans [17], this line of study has never been conducted in Chinese. Previous studies had showed that there may exist cross-cultural differences in HRQOL [19]–[20]. Thus, in the current study, we were interested in examining the domains and degrees of functioning and well-being that are affected by the frailty of elders residing in a community in Taiwan.

## Methods

### Population and participants

This was a population-based cross-sectional study. The target population consisted of all residents aged 65 and over in eight administrative neighborhoods of North District of Taichung City, Taiwan in June, 2009. Taichung is a city located in west-central Taiwan with a population of just over one million people, making it the third largest city on the island. The area of Taichung City is 163.4 sq km^2^, and its population density was 6,249/km^2^ in 2009. Taichung city consists of eight districts. There are a total of 36 administrative neighborhoods at North Districts and 214 administrative neighborhoods in Taichung City. The eight administrative neighborhoods in our study were all from North Districts. There were two reasons why these eight administrative neighborhoods were selected. One was that they were the administrative neighborhoods around our hospital and we planned to conduct a longitudinal study on this cohort in the future. Selecting these eight administrative neighborhoods would facilitate the follow-up in the future. The other reason was that all districts of Taichung City are of the same urbanization level. In addition, the age and gender distributions of these eight administrative neighborhoods are similar to those of Taichung population and Taiwan populations. There were a total of 3,997 elderly residents in these eight administrative neighborhoods during the time of the study, about 4.58% of the Taichung population of the same age. The sampling frame for this study was the set of all individuals' records from the Bureau of Households.

**Figure 1:**
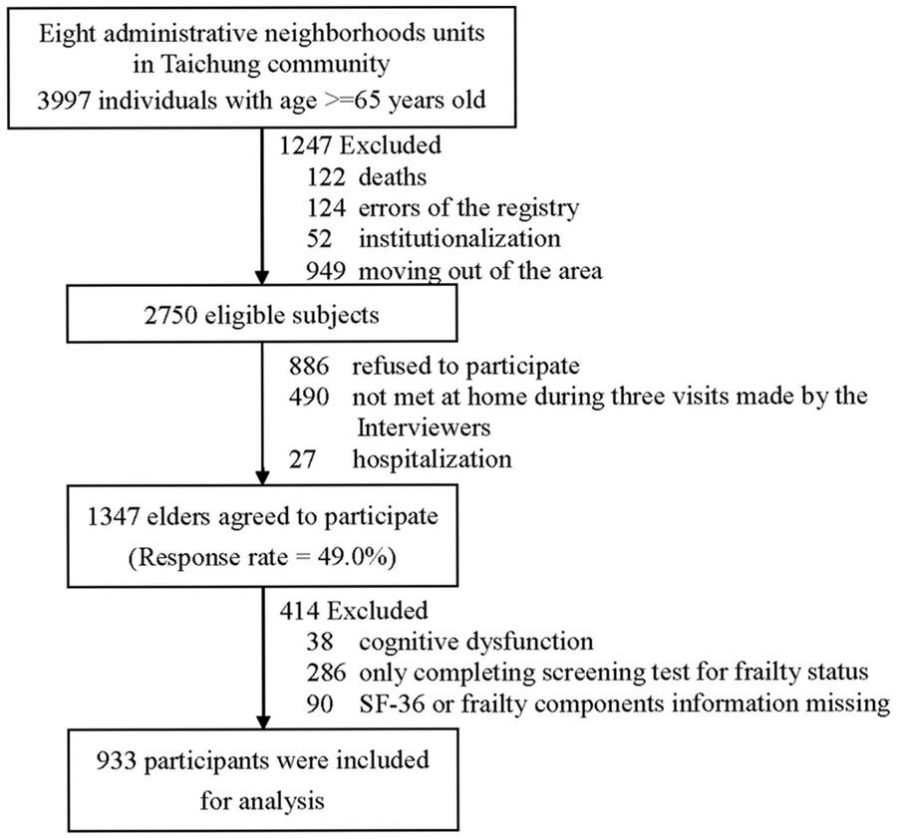
The flowchart of recruitment procedures of the current study.

All eligible individuals were invited to participate in the current study. Figure 1 shows the flowchart of recruitment procedures. During household visits, we identified 1,274 individuals who were not eligible and excluded them from the study sample. The reasons for exclusion included death (n = 122), institutionalization (n = 52), moving out of the area (n = 949), and errors of the registry (n = 124). A total of 2,750 subjects were eligible, and 1,347 agreed to participate and provide complete information. Thus, the overall response rate was 49.0%. This study was approved by the Human Research Committee of China Medical University Hospital. Written informed consent was obtained from each participant. Among these participants, 286 elders completed only the first stage of the screening test that included assessment of frailty measures and did not fill out the SF-36 questionnaires. A total of 933 elders were included in the current data analysis after excluding those diagnosed as dementia (n = 21), without Mini-Mental scores (MMSE) information (n = 11), with MMSE were less than 14 points (n = 6), and those who had incomplete frailty-related components or SF36 information (n = 90).

### Measurements

#### Frailty measures

Frailty was defined on the basis of well established, standardized and widely accepted phenotype described by Fried et al. in the Cardiovascular Health Study [3]. It composed of 5 components: shrinking, weakness, poor endurance and energy, slowness, and low physical activity level. Four of five frailty components were exactly the same as those proposed by Fried. Only weight loss was adapted. Shrinking was defined as unintentional weight loss of≥3 kilograms in the prior year. Weakness was defined as grip strength in the lowest quintile at baseline, based on subgroups of gender and body mass index [3]. Poor endurance and energy were measured by self-reported exhaustion, identified by two questions from the Center for Epidemiological Studies-Depression scale [21]. Slowness was measured by the slowest quintile of the population based on the time needed to walk 15 feet, based on subgroups of gender and standing height [3]. Low physical activity level was measured by a weighted score of kilocalories expended per week based on each participant's report. The lowest quintile of physical activity in our study sample was identified for each gender.

Those with none of the above components were considered as robust, whereas those with one or two components were considered as pre-frail and those with more than two components as frail.

#### SF-36

The SF-36 is a short questionnaire with 36 items which measure eight multi-item variables: physical functioning (PF, 10 items), social functioning (SF, 2 items), role limitations due to physical problems (RP, 4 items), role limitations due to emotional problems (RE, 3 items), mental health (MH, 5 items), vitality (VT, 4 items), pain (BP, 2 items), and general perception of health (GH, 5 items). For each variable item, scores are coded, summed, and transformed to a scale from 0 (worst possible health state measured by the questionnaire) to 100 (best possible health state). In addition, the SF-36 Physical Component Summary (PCS) and the Mental Component Summary (MCS) scales are derived following the standard SF-36 scoring algorithms [22]. For the SF-36, a high score indicates a better state of health.

#### Other measures

Data on smoking, alcohol drinking and physical activity were collected by questionnaire when the participants underwent a complete physical examination. Smoking and alcohol drinking were dichotomized into two groups. Those in the non-smoking group had never smoked or had smoked less than 100 cigarettes during their lifetime, whereas those in the smoking group smoked currently or had smoked more than or equal to 100 cigarettes during their lifetime. Individuals who self-reported drinking alcohol or exercising were classified into the group with this specific characteristic. There are two additional questions with a binary response that measure the pain problem and sleep impairment.

### Statistical Analysis

Simple descriptive analyses, such as mean, standard deviation, proportion, Chi-square test, and t-test, were employed to analyze data when appropriate. Analysis of covariance (ANCOVA) was used to compare global group differences in SF-36 scales after age or multivariate adjustment.

In order to examine the relative burden of frailty status on the scales, comparisons of partial F-values of ANCOVA across eight scales were made. The method used for this assessment was derived from the concept of statistical efficiency [23]-[25]. A measure is more efficient, relative to another, if it yields a higher ratio of systematic variation relative to random variation. When we hold the sample size constant within comparisons of eight scales, the relative precision of these scales can be detected by comparing the magnitude of the F statistic (ratio of systematic variance relative to error variance) [24].

The multiple linear regression model was used to test the independent effects of frailty components on physical functioning and well-being by controlling for the other independent variables. Regression models estimated the effects of frailty components on HRQOL (SF-36) by comparing elders with frailty components to elders screened as being without these components.

## Results

Of the 933 elders, 92 (9.86%), 415 (44.48%) and 426 (45.66%) were categorized as frail, pre-frail and robust, respectively. The distributions of demographic factors, chronic disease/condition, and behavior status for these three groups are compared in Table 1. Those who were pre-frail and frail were older, more likely to have an educational attainment of less than or equal to 6 years, and less likely to be married, had a higher prevalence of hypertension, diabetes, heart disease, stroke, Parkinson's disease, depression, cataract, pain problem, and sleep impairment, and were less likely to have regular exercise, smoke, and drink alcohol.

**Table 1:** Characteristics of subjects according to frailty status.

	Robust		Pre-frail		Frail		P value†
Variable	n	%	n	%	n	%	
All	426	45.66	415	44.48	92	9.86	--
***Demographic factor***							
Gender							0.109
Men	239	56.10	203	48.92	47	51.09	
Women	187	43.90	212	51.08	45	48.91	
Age							<0.001
≤70	205	48.12	133	32.05	15	16.30	
70–75	119	27.93	98	23.61	13	14.13	
>75	102	23.94	184	44.34	64	69.57	
Education							<0.001
Illiterate	29	6.97	63	15.56	19	21.59	
≤6 years	93	22.36	120	29.63	23	26.14	
7–12 years	164	39.42	129	31.85	23	26.14	
≥ 13 years	130	31.25	93	22.96	23	26.14	
Marital status							0.004
Married	318	74.65	290	69.88	52	56.52	
Others	108	25.35	122	29.40	39	42.39	
***Chronic disease/condition***							
Hypertension	196	46.45	237	57.66	56	61.54	0.001
Diabetes	51	12.14	81	19.66	25	27.17	<0.001
Heart disease	111	26.30	124	30.17	37	40.66	0.023
Kidney failure	4	0.95	9	2.21	2	2.22	0.323
Stroke	9	2.16	22	5.39	18	20.00	<0.001
Parkinson	2	0.48	11	2.68	4	4.40	0.010
Depression	7	1.67	12	2.93	8	8.79	0.001
Cataract	168	39.72	211	51.21	53	57.61	<0.001
Pain problem	196	47.69	226	56.64	63	70.79	<0.001
Sleep impairment	154	36.84	197	48.17	50	54.35	<0.001
***Behavior status***							
Regular exercise	360	84.71	297	71.74	33	36.26	<0.001
Smoking							0.007
No	341	80.05	328	79.04	63	68.48	
Current	42	9.86	40	9.64	7	7.61	
Former	43	10.09	47	11.33	22	23.91	
Alcohol drinking							0.012
No	338	79.34	339	81.69	74	80.43	
Current	65	15.26	55	13.25	6	6.52	
Former	23	5.40	21	5.06	12	13.04	

Robust: 0 frail components present; pre-frail :1–2 frail components present; frail: ≥3 frail components present.

† P values were calculated by chi-square test.

Table 2 provides multivariate-adjusted means and standard errors of the robust, pre-frail and frail elders. In general, the robust elders reported significantly better health than did the pre-frail and frail elders on all scales, and the pre-frail elders reported better health than did the frail elders on all scales except RP, RE and MCS after multivariate adjustment. The significantly negative differences between the frail and robust elders ranged from 3.58 points for MCS to 22.92 points for PF. The differences between robust and pre-frail elders were much lower. Larger values for the F statistic indicated a better ability to discriminate between elders in the “best” and “worst” response categories for these scales. The F statistics for testing the differences between the adjusted mean scores of elders in different frailty groups were highest for the PF scale (F statistic = 56.46) and lowest for the RE scale (F statistic = 7.01). This indicates that the PF scale discriminates better than the RE scale among elders of different frailty groups.

**Table 2:** Adjusted means and standard errors of SF-36 according to frailty statuses.

	Robust_(1)_		Pre-frail_(2)_		Frail_(3)_		ANCOVA	Multiple post-hoc comparison
Variable	Adjusted‡ mean	SE	Adjusted‡ mean	SE	Adjusted mean	SE	*F* value 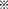 [Table-fn nt108]	
PF	85.66	1.32	80.62	1.25	62.74	1.96	56.46**	1>2>3
RP	93.95	2.67	83.26	2.52	78.16	3.96	12.13***	1>(2, 3)
BP	83.49	1.34	81.23	1.26	74.29	1.99	8.89***	1>2>3
GH	66.96	1.57	60.55	1.48	49.22	2.33	25.95***	1>2>3
VT	77.69	1.51	72.13	1.42	63.15	2.24	19.45***	1>2>3
SF	95.94	1.21	91.57	1.14	80.17	1.79	32.45***	1>2>3
RE	94.38	2.21	87.28	2.08	85.90	3.28	7.01***	1>(2, 3)
MH	82.31	1.33	79.69	1.25	73.67	1.97	8.17***	1>2>3
PCS	50.48	0.53	48.01	0.50	42.56	0.79	43.61***	1>2>3
MCS	56.22	0.62	54.47	0.59	52.64	0.92	8.10***	1>(2, 3)

Robust: 0 frail components present; pre-frail :1-2 frail components present; frail: ≥3 frail components present.

Physical functioning (PF), Role physical (RP), Bodily pain (BP), General health (GH), Vitality (VT), Social functioning (SF), Role emotional (RE), Mental health (MH), Physical component summary (PCS), Mental component summary (MCS).

‡ Adjusted for age, gender, education, marital status, chronic disease, pain problem, sleep impairment, regular exercise, smoking and drinking behaviors.

** p<0.01.

*** p<0.001; SE stands for standard error.

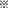
partial F value is presented.

Multiple regression analysis was used to simultaneously estimate the effects of frailty components in the eight scales and two summary scales of the SF-36 using multivariate adjustment (Table 3). In general, the estimated effects of the frailty components on all scales of the SF-36 were negative. Shrinking had a significant impact only on the RP of SF-36. Poor endurance and energy had a significant impact on all scales of the SF-36 except RP and RE. Low physical activity had a significant impact on PF, SF and PCS, which were scales of the primary physical component. Slowness had a significant impact on all scales, except RE, MH and MCS, and weakness had a significant impact on the PF, VT, SF, RE and MCS scales. In general, the magnitude of the effects of frailty components was largest for poor endurance and energy, and next for slowness. The percentages of the variations of these 10 scales explained by these factors ranged from 11.1% to 49.1%, with the lowest percentage for RE and the highest percentage for BP.

**Table 3:** The estimated parameters of five frailty components for 10 scales of SF-36.

	Estimate (standard error)									
	PF	RP	BP	GH	VT	SF	RE	MH	PCS	MCS
**Model**										
Shrinking	1.59	−7.76*	−1.62)	−2.31	0.01	−1.83	−2.53	−2.26	−0.74	−0.95
	(1.56)	(3.23)	(1.62	(1.89)	(1.79)	(1.47)	(2.75)	(1.63)	(0.63)	(0.77)
Poor endurance and energy	−9.82***	−6.93	−8.96***	−14.58***	−16.85***	−7.46***	−5.83	−7.79*	−4.63*	−3.85*
	(2.06)	(4.29)	(2.15)	(2.51)	(2.38)	(1.95)	(3.48)	(2.07)	(0.8)	(0.97)
Low physical activity	−15.59***	−6.22	−0.63	−2.68	3.09	−5.94***	−1.20	−0.61	−4.28*	1.37
	(2.21)	(4.60)	(2.31)	(2.69)	(2.55)	(2.09)	(3.87)	(2.3)	(0.88)	(1.08)
Slowness	−8.97***	−11.95***	−3.47***	−6.56***	−3.91***	−4.67***	−5.87	−0.92	−3.74*	−0.40
	(1.28)	(2.64)	(1.33)	(1.55)	(1.47)	(1.21)	(2.23)	(1.32)	(0.51)	(0.62)
Weakness	−4.37***	−0.04	0.11	−1.78	−3.75***	−3.46***	−4.30*	−1.10	−0.84	−1.24*
	(1.25)	(2.60)	(1.30)	(1.52)	(1.51)	(1.18)	(2.17)	(1.29)	(0.50)	(0.61)
*R^2^*	*44.4%*	*13.2%*	*49.1%*	*26.8%*	*23.3%*	*18.0%*	*11.1%*	*16.0%*	*47.1%*	*13.4%*

Physical functioning (PF), Role physical (RP), Bodily pain (BP), General health (GH), Vitality (VT), Social functioning (SF), Role emotional (RE), Mental health (MH), Physical component summary (PCS), Mental component summary (MCS).

Adjusted for age, gender, education, marital status, chronic disease, pain problem, sleep impairment, regular exercise, smoking and drinking behavior.

* p<0.05.

** p<0.01.

*** p<0.001.

**Figure 2:**
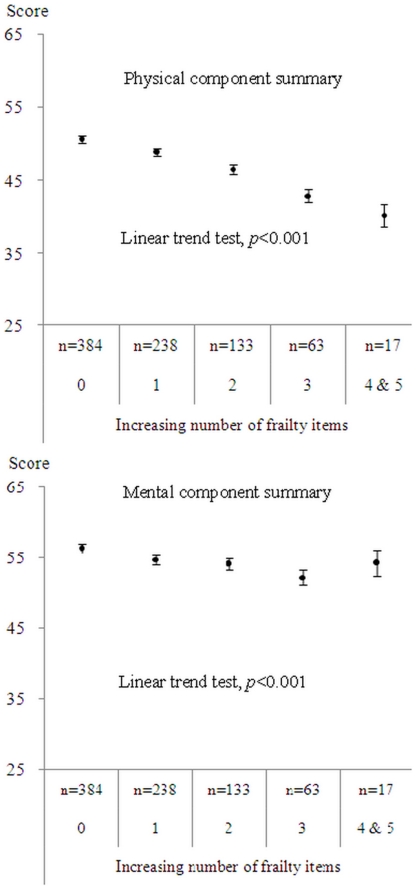
Relationship between physical and mental component summary and the frailty index. All values were adjusted for age, gender, education, marital status, chronic disease, pain problem, sleep impairment, regular exercise, smoking and drinking behavior. Adjusted mean and standard error are shown as circle point and vertical bar

Figure 2 shows the multivariate-adjusted means of the PCS and MCS of the SF-36 in elders, based on the number of frailty components. There was a linear decrease in the adjusted means of the PCS and MCS with the increasing number of frailty components (P for trend <0.001 for both the PCS and MCS), although there was a slight increase in the adjusted means of the MCS for the number of frailty components greater than or equal to four. A greater magnitude of reduction in the PCS than in the MCS was observed.

## Discussion

The purpose of this study was to assess the impact of frailty on functioning and well-being in elders residing in a community in central Taiwan. Frail elders reported significantly compromised HRQOL compared with elders without frailty in the same population. This demonstrates that frailty had a considerable impact, not only on the scales of the primary physical component, but also on the scales of the primary mental component. Most of the effects are both statistically and clinically significant if one accepts that differences of three to five points are considered clinically meaningful [11]. Our results showed that elders with frailty had noticeably negative effects on the eight scales of the SF-36, ranging from 8.64 (MH) to 22.92 (PF) points below the scores for elders without frailty. Of the five frailty components, poor endurance and energy exerted the greatest effects and slowness the next greatest.

Our results are in agreement with the findings of those studies that have examined the relationship between frailty and HRQOL, although different frailty definitions have been used. Similar to ours, Masel et al. adopted a modified definition of frailty proposed by Fried and SF-36 was used as a quality of life measure [17]. They found that being pre-frail or frail was significantly associated with lower scores on all physical and mental health related quality of life scales than being non-frail in older Mexican American individuals. Bilotta et al. found a negative trend in HRQOL with frailty, as measured by the Study of Osteoporotic Fractures for the dimensions of health, independence, home and neighborhood, psychological and emotional well-being, and leisure, activities and religion, in a cross-sectional study of 239 community-dwelling outpatients referred to a geriatric medicine clinic [13]. Buck et al. found an extra 13% of the variance in HRQOL was explained when the frailty index, developed by weighting age, number of comorbid conditions, and symptom severity, was added into the model with known predictors [14]. In addition, the relationship between grip strength, one of frailty components, and HRQOL was evaluated with 2,987 community-dwelling men and women aged 59-73 years of age, and the association was independent of age, size, physical activity and co-morbidity [26].

The reduction in HRQOL associated with frailty was higher in magnitude than that reported for chronic physical illnesses such as low back pain, arthritis, and diabetes [27], which implicates the severe impact of frailty. For instance, the negative effect of frailty on physical functioning in this study was −22.92, which was much worse than the impact of diabetes (−6.3) [27], while the negative effect of frailty on general perception was 17.74 points, which was also higher than the impact of diabetes, back pain, hypertension and stroke (about 15 points) [27].

A number of limitations should be noted in interpreting the results of this study. One is that the cross-sectional design of the study does not allow for any prospective conclusion on the relationship of frailty with HRQOL. Second, the sample was selected from a Taiwanese metropolitan elderly population, thus our results may not be applied to those elders of rural areas. Third, because only elders residing in community were studied, our results may not be representative of elders in institutions. Last, the response rate was 49.0%. A small proportion of people was hospitalized and these hospitalized elders were more likely to be frail. Therefore it may lead to an underestimation of the frailty prevalence. By contrast, a greater proportion of people not met at home are possibly less frail than the average which may result in an overestimation of the frailty prevalence in the population. Similarly, 30.7% of the elders who agreed to participate in the study did not to fill out the SF-36 questionnaires or had missing data, indicating that potential missing bias might exist. Due to non-response or incomplete data, some degree of selection bias cannot be excluded.

Despite these limitations, the present study is the first to examine the impact of frailty on function and well-being in community-dwelling elders. The SF-36 measures functional status, well-being, and overall health, which are of prime concern to patients, and it provides yardsticks for HRQOL. We illustrated the profiles of HRQOL for elders with frailty contrasted to those without frailty. Examining the association between frailty and HRQOL facilitates understanding about the meaning of differences between generic health measures scale scores and the clinical measures that are familiar to clinicians.

Our study results demonstrated that the differences in HRQOL between elders with and without frailty were substantial, and frailty might account for the differences. Future studies exploring the longitudinal relationship between frailty and HRQOL should be conducted to further clarify the causal relationship between frailty and HRQOL.

### Conclusion

Our study demonstrates that the disabilities in physical health inherent in frailty can be linked to a reduction in HRQOL as measured by the SF-36. Such an association between clinical measures and a generic measure of HRQOL may offer clinicians new information to understand frailty and to conceptualize it within the broader context of disability. The reduction in HRQOL of elders with frailty could have clinical and health management consequences and merits further study.
